# *lin-41* controls dauer formation and morphology via *lin-29* in *C. elegans*

**DOI:** 10.17912/micropub.biology.000323

**Published:** 2020-11-12

**Authors:** Allison R Cale, Xantha Karp

**Affiliations:** 1 Department of Chemistry and Biochemistry, Central Michigan University, Mount Pleasant, MI 48859; 2 Current address: Department of Human Genetics, University of Michigan, Ann Arbor, MI 48109; 3 Department of Biology, Central Michigan University, Mount Pleasant, MI 48859

**Figure 1:**
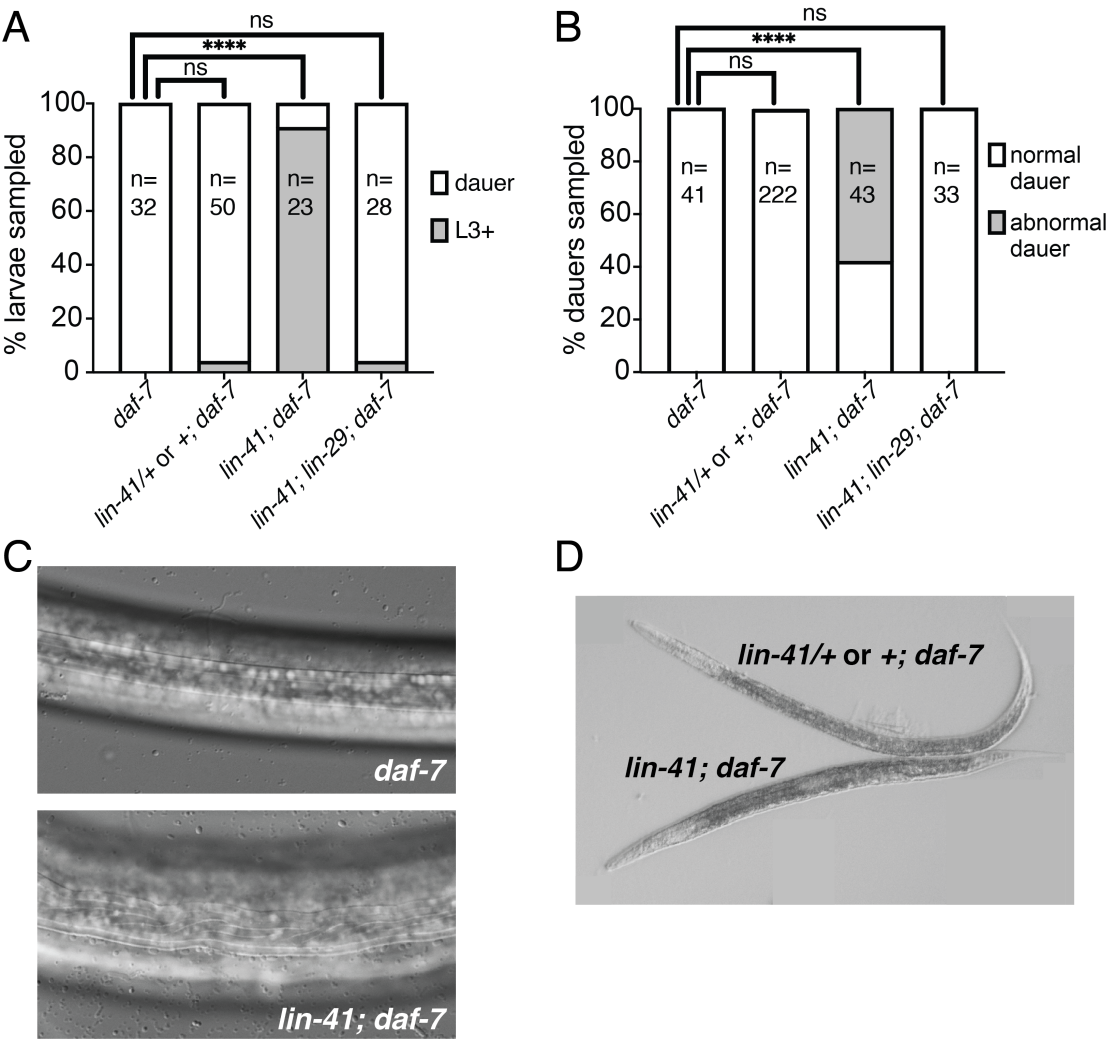
***lin-41(0); daf-7* mutants display a *lin-29-*dependent dauer defective phenotype:** (A) The *daf-7(e1372)* allele was used to promote dauer entry, and was present in all strains. Larvae homozygous for the *lin-41* null allele *n2914 (“lin-41(0)”)* rarely entered dauer at the dauer-inducing temperature of 24˚C, in comparison to control larvae that were wild-type for *lin-41* or heterozygous for *lin-41(0). lin-41(0)* was balanced with the closely-linked transgene *nIs408[LIN-29::mCherry, ttx-3::gfp]* (Harris and Horvitz 2011)*. lin-41(0)* homozygous larvae were identified by lack of fluorescence, whereas *lin-41(0)/+* or *+* refers to larvae that were positive for fluorescence. Simultaneous loss of *lin-29 (“lin-29(0)”)* suppressed the dauer-defective phenotype of *lin-41(0)*, indicating that misregulation of *lin-29* inhibits dauer entry in *lin-41(0)* larvae. (B) SDS-selection from starved plates yielded *lin-41(0)* homozygousdauer larvae, over half of whichdisplayed morphological defects. Morphological defects were absent in *lin-41(0); lin-29(0)* dauers from starved plates, indicating that misregulation of *lin-29* can result in abnormalities in dauer morphology. (C) *daf-7* dauer larvae (top) from starved plates displayed distinct dauer alae with crisp, parallel lines as well as the long, thin body shape characteristic of dauer larvae. By contrast, some *lin-41(0)* dauer larvae displayed morphological defects including crooked and convergent alae and a lumpy body shape (bottom). Images were taken using a 63X objective. (D) Control *lin-41(0)/nIs408* or *nIs408* dauer larvae were long and thin, whereas some *lin-41(0)* homozygous dauer larvae appeared Dpy. Images were taken using a 10X objective. Fisher exact test: ns, not significant; ****, P<0.0001.

## Description

In response to adverse environments *C. elegans* larvae may form stress-resistant and developmentally arrested dauer larvae. Entry into dauer interrupts developmental progression after the second larval molt (Cassada and Russell 1975), and the dauer formation decision must be coordinated with developmental pathways. Heterochronic genes control stage-specific cell fate decisions, and these genes interconnect with the dauer formation *(daf)* pathway in several ways. Dauer formation and *daf* genes affect heterochronic phenotypes, and conversely, heterochronic genes can impact dauer by controlling the timing of dauer formation, the morphology of dauer larvae, or the ability to enter dauer (Liu and Ambros 1989; Liu and Ambros 1991; Antebi *et al.* 1998; Tennessen *et al.* 2010; Karp and Ambros 2011; Karp and Ambros 2012). Here we find that the *lin-41* heterochronic gene regulates the dauer formation decision and dauer morphogenesis via its canonical target, *lin-29.*

The temperature-sensitive *daf-7(e1372)* allele results in constituitive dauer entry at 25˚C, and the allele is a useful tool for controlling dauer entry (Vowels and Thomas 1992; Karp 2018). While performing experiments examining the role of *lin-41* during dauer, we observed that *lin-41(0); daf-7* worms rarely entered dauer. To better understand this phenotype, we quantified the number of dauer and nondauer larvae in synchronous populations of *daf-7(e1372)* larvae grown at the dauer-inducing temperature of 24˚C.

*lin-41(0)* mutants are sterile, and therefore the strain must be propagated as a heterozygote (Slack *et al.* 2000). We took advantage of a closely linked transgene, *nIs408[LIN-29::mCherry, ttx-3::gfp]* to balance *lin-41(0)* (Harris and Horvitz 2011)*.* *lin-41(0)* homozygous larvae were identified by the absence of fluorescence. Controls included *nIs408-*positive siblings, 2/3 of which should be *lin-41(0)/nIs408* and 1/3 of which should be *nIs408/nIs408,* as well as a strain that is homozygous for the wild-type allele of *lin-41.*

Embryos from each strain were incubated at 24˚C for 50-52 hours to induce dauer formation. As expected, 100% of larvae in the control *daf-7* strain were dauers (Fig. 1A). Similarly, the *lin-41(0)/nIs408* or *nIs408/nIs408; daf-7* larvae formed dauers at high penetrance (96%). By contrast, only 2/23 (9%) of *lin-41(0)* homozygous larvae were in dauer. The remaining *lin-41(0)* larvae had continued development and were in the L3 or L4 stage (Fig. 1A). Therefore, *lin-41(0)* larvae display a dauer formation-defective (Daf-d) phenotype.

The canonical role for *lin-41* as a heterochronic gene is to promote larval cell fate in hypodermal cells by directly repressing translation of *lin-29* (Slack *et al.* 2000; Aeschimann *et al.* 2017)*. lin-29* encodes a transcription factor that promotes adult cell fate (Rougvie and Ambros 1995). In addition to this canonical role, *lin-41* controls several other processes, including oocyte growth and maturation, male tail tip morphogenesis, and a sex-specific neurotransmitter switch in the AIM interneurons, among others. *lin-41* acts via *lin-29* to control some of these processes, and via other targets to control other processes (Del Rio-Albrechtsen *et al.* 2006; Spike *et al.* 2014; Pereira *et al.* 2019).

To ask whether *lin-29* is required for the Daf-d phenotype we observed in *lin-41(0)* mutants, we examined *lin-41(0); lin-29(0)* larvae segregating from *lin-41(0)/nIs408; lin-29(0)* mothers. Because the *nIs408* transgene rescues *lin-29(0)* (Harris and Horvitz 2011)*,* this transgene effectively balances both *lin-41* and *lin-29.* We found that the additional loss of *lin-29(0)* suppressed the *lin-41(0)* dauer entry-defective phenotype, as 96% of *lin-41(0); lin-29(0); daf-7* larvae examined were in dauer (Fig. 1A).

Since obtaining *lin-41(0)* dauers using the *daf-7* method was inefficient, we asked whether utilization of the natural starvation response could drive *lin-41(0); daf-7* larvae into dauer. We allowed each strain to crowd and starve out, and then selected for dauer larvae using 1% SDS (Cassada and Russell 1975). We were able to obtain SDS-resistant *lin-41(0)* homozygous dauer larvae using this method. Forty-two percent of *lin-41(0)* dauer larvae displayed normal dauer morphology, as did the two dauer larvae obtained from *daf-7* induced dauer formation (Fig. 1A). Interestingly, the remaining 58% of *lin-41(0)* dauer larvae displayed defective dauer morphology (Fig. 1B). Specifically, both dauer alae and body shape were abnormal. In contrast to the straight ridges that make up wild-type dauer alae, *lin-41(0)* mutant dauers displayed wavy dauer alae (Fig. 1C). With respect to body shape, there was a range in severity from worms of a normal length but a somewhat lumpy appearance to Dpy dauers that were shorter and fatter than controls (Fig. 1D).

These dauer morphology defects are reminiscent of those caused by loss of either of two other heterochronic genes: *lin-14* or *lin-28* (Liu and Ambros 1989)*.* However, those defects appear to be due to areas of lateral cuticle that lack dauer alae. Interestingly, the *lin-28(0)* defect is suppressed by loss of *lin-29,* whereas the *lin-14* defect is not (Liu and Ambros 1989). By contrast, we never observed gaps in dauer alae in *lin-41(0)* mutants. Furthermore, similarly to the Daf-d phenotype, we found that the dauer morphology phenotype observed in the *lin-41(0)* homozygotes was suppressed by loss of *lin-29,* as no *lin-41(0); lin-29(0); daf-7* dauer larvae from the SDS experiments displayed defective dauer morphology (Fig. 1B).

In summary, *lin-41* regulates two aspects of dauer biology, the decision to enter dauer, and dauer morphogenesis. In both cases, *lin-41* appears to act via its canonical target, *lin-29.* This finding adds to our understanding of the apparent coordination between dauer formation pathways and heterochronic pathways.

## Methods

**Strains and maintenance.**

*C. elegans s*trains were maintained at 20˚C on NGM plates with *E. coli* strain OP50 as a food source (Brenner 1974).

**Dauer-induction via *daf-7* and dauer formation assay**

Individual gravid adult hermaphrodites from each strain were transferred to 60mm NGM plates and allowed to lay embryos for 3-5 hours at 24**˚**C. Adults were then removed, and embryos were incubated at 24**˚**C for an additional 50 to 52 hours. For heterozygous strains, any plates containing homozygous mothers were discarded. Larvae were then imaged as described below. For strains XV238 *(lin-41/nIs408; daf-7)* and the control VT1777 *(daf-7),* every larva on the plate was scored for developmental stage. For strain XV239 *lin-41/nIs408; lin-29; daf-7),* only worms that lacked *nIs408* were scored, since *nIs408* balances *lin-41* and rescues *lin-29.* Non-dauer larvae were distinguishable from dauer larvae primarily due to the lack of dauer alae, in addition to their larger size, lack of radial constriction, and progression of gonad and vulval development. Six independent trials were performed.

**Dauer induction via starvation and dauer morphology assay**

Strains were propagated at 20˚C on 60mm NGM plates seeded with OP50. Heterozygous strains were maintained by cloning out individuals and verifying the correct segregation of phenotypes in the progeny. Plates were monitored to identify the point at which the food was exhausted and the point at which dauer larvae appeared. Sets of XV238, XV239, and VT1777 populations that starved at the same time were paired for experiments, to ensure that dauer larvae were approximately the same age between strains within a single experiment. The data in Figure 1B represent 6 independent trials. Dauer larvae were selected using a 30-35 minute incubation in 1% SDS at room temperature. For strain XV239, only larvae lacking fluorescence were analyzed.

**Microscopy**

Larvae were picked to slides with 2% agarose pads and anesthetized in 0.1 M levamisole. Worms were visualized using a Zeiss AxioImager D2 compound microscope with DIC optics. Representative images acquired using an AxioCam MRm Rev 3 camera and Zen software (2012) from Zeiss are shown. Brightness/contrast was adjusted using Adobe Photoshop.

## Reagents

**Strains used in this study**

**Table:** 

Strain name	Genotype
VT1777	daf-7(e1372); maIs105[col-19::gfp]
XV238	lin-41(n2914)/nIs408[LIN-29::mCherry, ttx-3::gfp]; daf-7(e1372); maIs105
XV239	lin-41(n2914)/nIs408; lin-29(n546); daf-7(e1372); maIs105
